# Energetic performance index improvement after Glenn and Damus-Kaye-Stansel procedure using vector flow mapping analysis: a case report

**DOI:** 10.1186/s40981-020-0312-4

**Published:** 2020-01-21

**Authors:** Atsushi Kainuma, Koichi Akiyama, Yoshifumi Naito, Kazuma Hayase, Hisayuki Hongu, Keiichi Itatani, Masaaki Yamagishi, Teiji Sawa

**Affiliations:** 1 Department of Anesthesiology, Kyoto Prefectural University of Medicine, 465 Kajii-cho, Kamigyoku, Kyoto, 602-8566 Japan; 2 Department of Anesthesiology, Yodogawa Christian Hospital, 1 Chome-7-50, Kunijima, Higashiyodogawa Ward, Osaka, 533-0024 Japan; 3 Department of Anesthesia and Perioperative care, University of California San Francisco, 505 Parnassus Ave, San Francisco, CA 94143 USA; 4 Department of Pediatric Cardiovascular Surgery, Children’s Medical Center, Kyoto Prefectural University of Medicine, 465 Kajii-cho, Kamigyoku, Kyoto, 602-8566 Japan; 5 Department of Cardiovascular Surgery, Kyoto Prefectural University of Medicine, 465 Kajii-cho, Kamigyoku, Kyoto, 602-8566 Japan

**Keywords:** Vector flow mapping, Damus-Kaye-Stansel procedure, Glenn procedure, Perioperative echocardiography, Energetic performance index

## Abstract

**Background:**

Echocardiography vector flow mapping can assess dynamic flow to treat congenital heart diseases. We evaluated intracardiac flow, energy loss, left ventricular output kinetic energy, and energetic performance index using vector flow mapping during Glenn and Damus-Kaye-Stansel procedures in order to assess the efficacy of the surgery.

**Case presentation:**

A 9-month-old boy underwent Glenn and Damus-Kaye-Stansel procedures. The energy loss depends on the left ventricular preload; therefore, energy loss decreased after the Glenn procedure. After the Damus-Kaye-Stansel procedure, the kinetic energy would increase owing to the integrated systemic outflow; however, in our case, kinetic energy decreased, which was potentially explained by the fact that kinetic energy also depends on the left ventricular preload. After the Glenn and Damus-Kaye-Stansel procedures, we detected an improvement in energetic performance index, indicating that the cardiac workload improved as well.

**Conclusion:**

We revealed the efficiency of the Glenn and Damus-Kaye-Stansel procedures using vector flow mapping.

## Background

Echocardiography vector flow mapping (VFM) can visualize blood flow and calculate energy loss (EL) in the left ventricle [1]. This flow visualization technique can assess dynamic flow to treat congenital heart diseases [2, 3]. However, there have been few reports evaluating EL during congenital heart surgery [4, 5]. In this case, we evaluated intracardiac flow, EL, and left ventricular output kinetic energy (KE) using VFM during the Glenn and Damus-Kaye-Stansel (DKS) procedures in order to assess the efficacy of the surgery. We have reported how to interpret these new indices in the perioperative period.

## Case presentation

A 9-month-old boy was diagnosed with a single left ventricle with a hypoplastic right ventricle, double inlet left ventricle, and ostium secundum defects at birth. At the age of 2 months, he underwent pulmonary artery banding to optimize pulmonary blood flow. At the age of 7 months, upon catheterization examination, his pulmonary artery pressure was 14 mmHg, pulmonary artery vascular resistance was 0.80 unit m^2^, and pulmonary artery index was 366 mm^2^/m^2^. At the age of 9 months, he was scheduled for elective Glenn and DKS procedures.

**Fig. 1:**
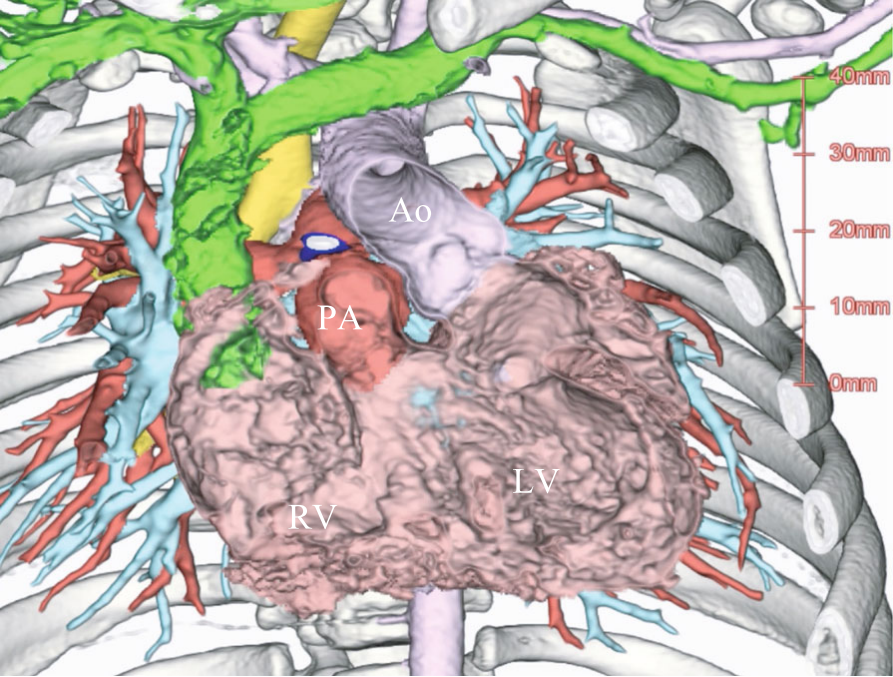
Preoperative computed tomography image showing the patient’s anatomical condition. Abbreviations: Ao, aorta; PA, pulmonary artery; RV, hypoplastic right ventricle; LV, left ventricle

On admission, his height, weight, and body surface area (BSA) were 66 cm, 6.1 kg, and 0.32 m^2^, respectively. His oxygen saturation was 85% in room air in the operation theater. Preoperative computed tomography showed the anatomical condition (Fig. 1). Anesthesia induction was uneventful, and transesophageal echocardiography (TEE) was performed during pre- and post-cardiopulmonary bypass (CPB); the patients’ vital signs were stable. The stored ultrasound images from the Prosound F75 Premier (Hitachi Aloka Medical, Tokyo, Japan) were transferred to a computer for analysis with the VFM software (DAS-RS-1, Hitachi-Aloka Medical, Tokyo, Japan). We evaluated intracardiac flow, EL, KE, and the ratio of the KE cycle to EL cycle (energetic performance index, EPI).

**Fig. 2:**
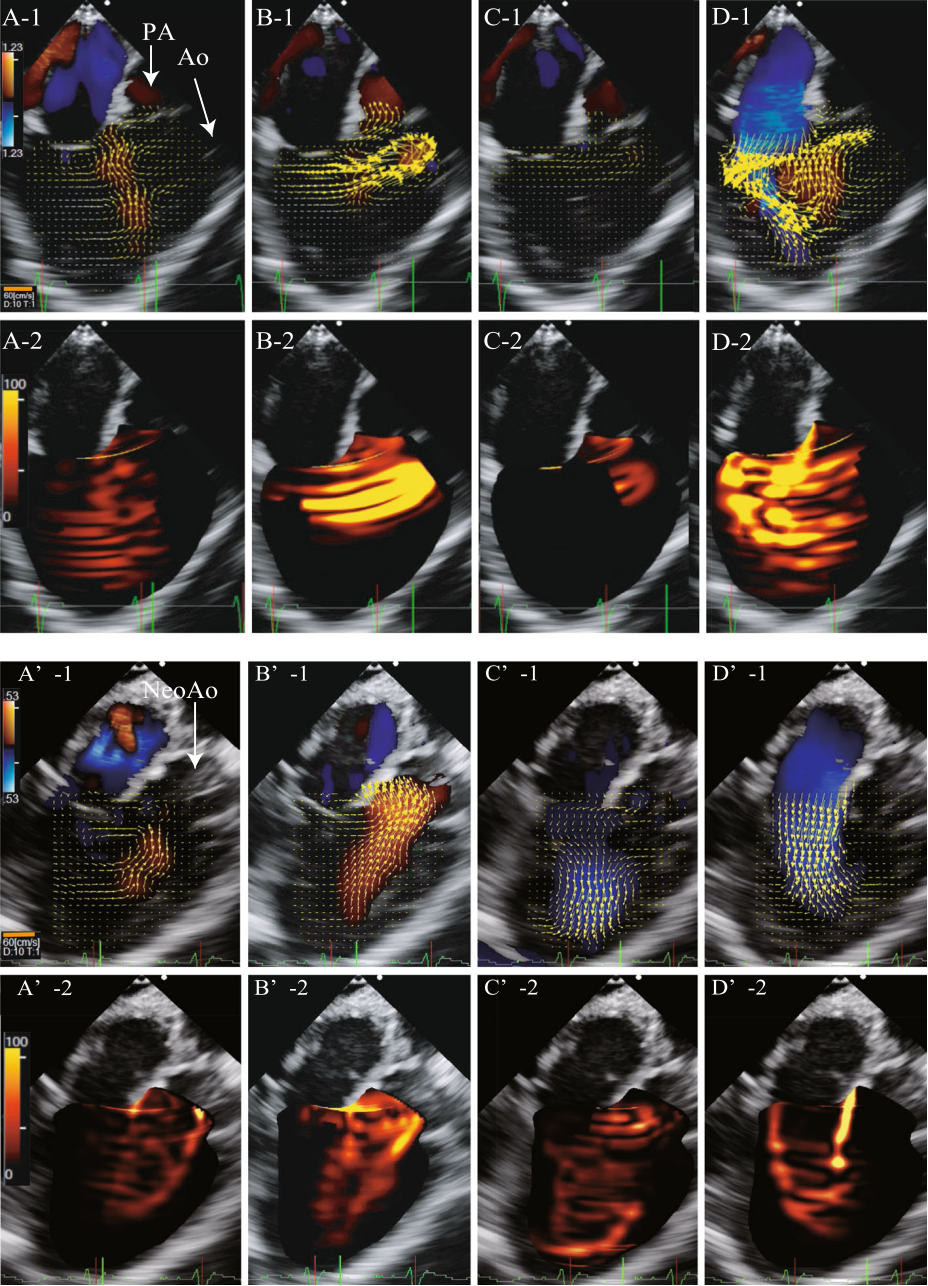
Vector flow mapping by transesophageal echocardiography. Vector flow mapping (VFM) of the mid-esophageal long-axis view before (A–D) and after (A'–D') cardiopulmonary bypass. Brightness indicates high energy loss. After the surgery, EL decreased dramatically, especially in the diastolic phase. A,A’:isovolumic contraction phase. B,B’:systolic phase. C,C’:isovolumic relaxation phase. D,D’:Diastolic phase. -1: vector flow mapping image, orange line is scale bar of the vector. -2: Energy loss image. Abbreviations: Ao, aorta; EL, energy loss; Neo Ao, neo aorta; PA, pulmonary artery

**Fig. 3:**
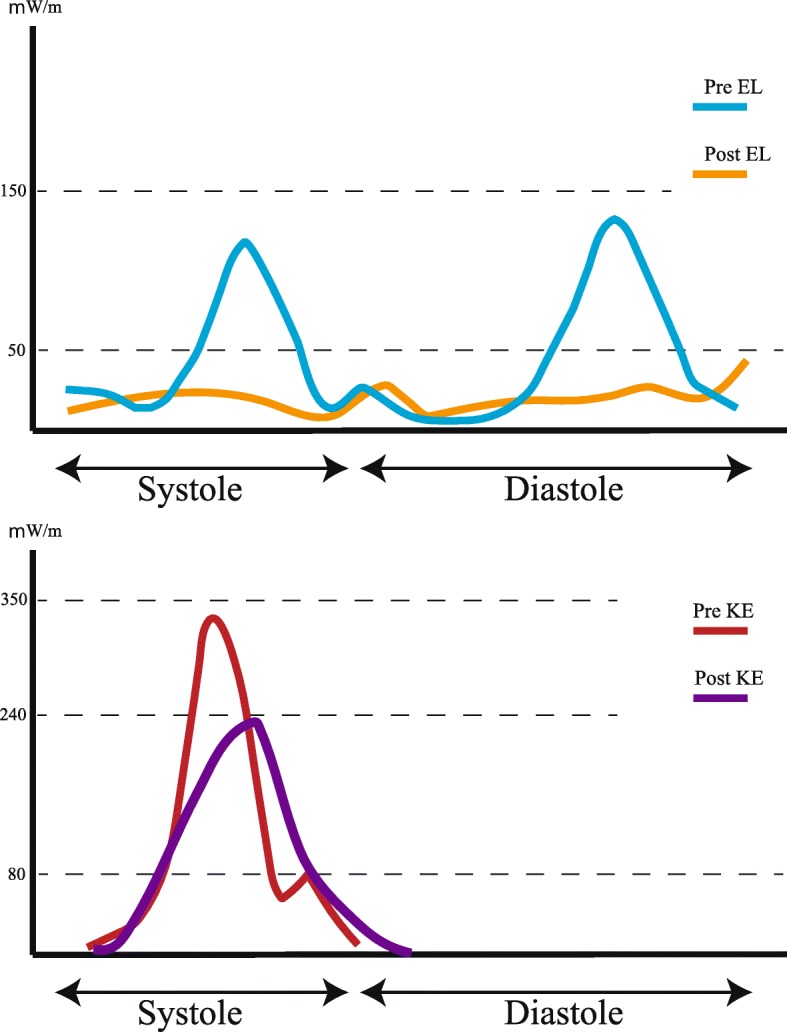
Flow energy loss (top) and flow kinetic energy (bottom) during one cardiac cycle. Upper graph demonstrates the pattern of flow energy loss during one cardiac cycle. The blue line presents preoperative energy loss, and the yellow line represents postoperative energy loss. Lower graph demonstrates the pattern of flow kinetic energy of the left ventricle during one cardiac cycle. The red line presents preoperative kinetic energy, and the purple line represents postoperative energy loss

We performed Glenn, DKS, and atrial septal defect enlargement procedures. Pre- and post-CPB hemodynamic parameters are shown in Table 1. Figure 2 shows the pre- and post-CPB VFM images analyzed by TEE, and Fig. 3 shows the pre- and post-CPB chart of EL, and KE values during one cardiac cycle. Furthermore, Additional file 1: Movie S1 and Additional file 2: Movie S2 show the pre-CPB VFM and EL and Additional file 3: Movie S3 and Additional file 4: Movie S4 show the post-CPB VFM and EL in the mid-esophageal long-axis views by TEE, respectively. After the surgery, the mean EL and mean KE decreased from 39.8 to 14.5 mW/m and from 49.7 to 46.5 mW/m, respectively. EPI increased from 1.25 to 3.20. Consequently, the patient was moved to the general ward 23 days after the initial surgery, without any inotropic support.

**Table 1:** Pre- and post-cardiopulmonary bypass hemodynamic parameters

	Pre-CPB	Post-CPB
HR (bpm)	133	151
BP (mmHg)	105/58	91/48
SpO_2_ (%)	95	76
FiO_2_	0.33	0.98
CVP (mmHg)	12	11

*BP* blood pressure, *CPB* cardiopulmonary bypass, *CVP* central venous pressure, *FiO*_*2*_ fraction of inspired oxygen, *HR* heart rate, *SpO*_*2*_ peripheral capillary oxygen saturation

## Discussion and conclusion

EL and KE are new indices and can be measured using VFM technology in the perioperative period. However, there are some clinical concerns about interpreting these indices. In this case, we used the EPI to overcome the limitation and interpret EL, KE, and the efficacy of the surgery appropriately.

VFM technology uses both color Doppler images and speckle tracking images [1]. Intracardiac EL can be calculated using the following equation:

$\mathrm{Energy}\ \mathrm{loss}\ \left(\mathrm{EL}\right)=\int \mu \left(2{\left(\frac{\partial u}{\partial x}\right)}^2+{\left(\frac{\partial v}{\partial y}\right)}^2+{\left(\frac{\partial u}{\partial y}+\frac{\partial v}{\partial x}\right)}^2\right) dA$*,* where *μ* is the viscosity of the blood, *u* and *v* are the velocity components along the Cartesian axes (*x* and *y*, respectively), and *A* is the area of the unit of the grid. EL has also been estimated by using computer flow simulation studies to assess hemodynamics following congenital heart disease [6–8]. Echocardiography VFM enables us to evaluate the cardiac workload. Evaluation of pulmonary valve stenosis and association between right ventricular function deterioration and EL using VFM technology have been reported [3, 9]. One of the well-known concept of EL is the “energy loss concept,” which is used to evaluate aortic stenosis [10], and the energy loss index is one of the predictors of prognosis in asymptomatic aortic stenosis [11].

However, there are some important points to be considered in interpreting the EL value during anesthesia. During CPB, the EL is zero because the heart undergoes asystole and does not produce energy. In a patient with severe aortic regurgitation [12, 13], or when systolic anterior motion exists [5], the patient’s left ventricular EL increases, which is caused by vortex and turbulent flow. In a hyper-dynamic state [14], the EL rises even if the patient does not need any therapeutic intervention. Therefore, when using VFM analysis during the early perioperative period, we should consider not only whether is the EL rising or falling, but also the clinical background in which the condition occurs. EL evaluation is difficult, which is why there are few reports about the relationship between EL and early perioperative prognosis.

To solve this problem, Akiyama et al. reported the utility of KE and EPI [14].

$\mathrm{Kinetic}\ \mathrm{energy}\ \left(\mathrm{KE}\right)=\int \frac{1}{2}\rho {v}^2\times vdL$*,* where *ρ* is the density of the blood (1060 kg/m^3^), *v* is the velocity vector of the blood flow, and dL is an increment of the cross-sectional line. In this case, we calculated left ventricular output KE, because we could acquire optimal images from TEE. In our case, the EPI is defined as follows:

$$ \mathrm{Energetic}\ \mathrm{performance}\ \mathrm{index}\ \left(\mathrm{EPI}\right)=\frac{\mathrm{KEcycle}}{\mathrm{ELcycle}} $$

The EPI is useful for assessing the cardiac condition, effectiveness of treatment, and outcome of surgery [14, 15]. Nakashima et al. reported the energy efficiency ratio, which is the left ventricular EL divided by KE of the trans-mitral flow, to analyze the postoperative cardiac condition [16]. By considering energy efficiency as a ratio, it is easier to evaluate the cardiac condition.

Theoretically, after the Glenn procedure, blood flows from the superior vena cava directly into the pulmonary artery, and the patient’s left ventricular preload decreases [17, 18]. The mean EL depends on the left ventricular preload; therefore, after the procedure, the EL was decreased.

The mean kinetic energy depends on the ejection of blood from the left ventricle into the left ventricular outflow tract. After the DKS procedure, the KE would increase owing to the integrated systemic outflow; however, in this case, KE decreased, which was potentially explained by the fact that KE also depends on the left ventricular preload [14]. Interestingly, EL and KE were reported to decrease in an 11-month-old patient who underwent the Glenn procedure, which may be consistent with our findings [4]. Together, evaluation of EL and KE is difficult because the left ventricular preload changes dramatically during pediatric congenital heart surgery.

Thus, we tried to calculate the EPI. After the Glenn and DKS procedures, we detected an improvement in EPI, indicating that the cardiac workload improved as well. Using VFM technology, the efficiency of the congenital heart disease surgery can be assessed in the early postoperative period.

The systolic and diastolic EL were positively correlated with the heart rate, and the E wave peak velocity and negatively correlated with age [19]. In this case, EL/BSA is still higher than the reference value. If this patient undergoes Fontan surgery, cardiac preload will decrease and the EL will decrease appropriately. The EL measurement was two-dimensional in this case. Three-dimensional measurements using magnetic resonance imaging might allow a more accurate assessment of the ventricular workload [19]. Further research should be performed to analyze the blood flow changes between the Glenn and Fontan surgeries.

The VFM blood flow analysis has some advantages and limitations. We believe that intraoperative blood flow analysis can contribute to the understanding of the pediatric congenital heart disease physiology, thus helping in improving these patients’ prognosis. This report would be helpful and useful for anesthesiologists and surgeons to interpret perioperative blood flow analysis appropriately during congenital heart surgery.

In conclusion, we revealed the efficiency of the Glenn and DKS procedures using VFM. After Glenn and DKS procedures, we detected an improvement in EPI, with an appropriate improvement in the mean cardiac workload. The EPI might be a helpful index of the hemodynamic status in patients with a single ventricle.

## Supplementary information

**Additional file 1: Movie S1.** Vector flow mapping of the mid-esophageal long-axis view before cardiopulmonary bypass.

**Additional file 2: Movie S2.** Energy loss in the mid-esophageal long-axis view before cardiopulmonary bypass.

**Additional file 3: Movie S3.** Vector flow mapping of the mid-esophageal long-axis view after cardiopulmonary bypass.

**Additional file 4: Movie S4.** Energy loss in the mid-esophageal long-axis view after cardiopulmonary bypass.

## Data Availability

The datasets generated and analyzed in this study are available from the corresponding author on request.

## Abbreviations

Ao: Aorta
CPB: Cardiopulmonary bypass
DKS: Damus-Kaye-Stansel
EL: Energy loss
EPI: Energetic performance index
KE: Kinetic energy
Neo Ao: Neo aorta
PA: Pulmonary artery
TEE: Transesophageal echocardiography
VFM: Vector flow mapping

## References

[1] Itatani K, Okada T, Uejima T, Tanaka T (2013). Intraventricular flow velocity vector visualization based on the continuity equation and measurements of vorticity and wall shear stress. Jpn J Appl Phys.

[2] Honda T, Itatani K, Takanashi M, Kitagawa A, Ando H, Kimura S (2017). Exploring energy loss by vector flow mapping in children with ventricular septal defect: pathophysiologic significance. Int J Cardiol Heart Vasc..

[3] Honda T, Itatani K, Miyaji K, Ishii M (2013). Assessment of the vortex flow in the post-stenotic dilatation above the pulmonary valve stenosis in an infant using echocardiography vector flow mapping. Eur Heart J..

[4] Kinoshita M, Akiyama K, Itatani K, Yamashita A, Ishii M, Kainuma A (2017). Energetic performance analysis of staged palliative surgery in tricuspid atresia using vector flow mapping. Cardiovasc Ultrasound..

[5] Akiyama K, Naito Y, Kinoshita M, Ishii M, Nakajima Y, Itatani K (2017). Flow energy loss evaluation in a systolic anterior motion case after the Ross procedure. J Cardiothorac Vasc Anesth..

[6] Whitehead KK, Pekkan K, Kitajima HD, Paridon SM, Yoganathan AP, Fogel MA (2007). Nonlinear power loss during exercise in single-ventricle patients after the Fontan: insights from computational fluid dynamics. Circulation..

[7] Itatani K, Miyaji K, Qian Y, Liu JL, Miyakoshi T, Murakami A (2012). Influence of surgical arch reconstruction methods on single ventricle workload in the Norwood procedure. J Thorac Cardiovasc Surg..

[8] de Leval MR, Dubini G, Jalali FM, Jalali H, Camporini G, Redington A (1996). Use of computational fluid dynamics in the design of surgical procedures: application to the study of competitive flows in cavopulmonary connections. J Thorac Cardiovasc Surg..

[9] Shibata M, Itatani K, Hayashi T, Honda T, Kitagawa A, Miyaji K (2018). Flow energy loss as a predictive parameter for right ventricular deterioration caused by pulmonary regurgitation after tetralogy of Fallot repair. Pediatr Cardiol..

[10] Garcia D, Pibarot P, Dumesnil JG, Sakr F, Durand LG (2000). Assessment of aortic valve stenosis severity. Circulation..

[11] Bahlmann E, Gerdts E, Cramariuc D, Gohlke-Baerwolf C, Nienaber CA, Wachtell K (2013). Prognostic value of energy loss index in asymptomatic aortic stenosis. Circulation..

[12] Stugaard M, Koriyama H, Katsuki K, Masuda K, Asanuma T, Takeda Y (2015). Energy loss in the left ventricle obtained by vector flow mapping as a new quantitative measure of severity of aortic regurgitation: a combined experimental and clinical study. Eur Heart J Cardiovasc Imaging..

[13] Itatani K (2014). When the blood flow becomes bright. Eur Heart J.

[14] Akiyama K, Maeda S, Matsuyama T, Kainuma A, Ishii M, Naito Y (2017). Vector flow mapping analysis of left ventricular energetic performance in healthy adult volunteers. BMC Cardiovasc Disord..

[15] Akiyama K, Itatani K, Yamashita A, Sawa T (2017). Visualization of suppressed intraventricular flow by constrictive pericarditis. J Clin Anesth..

[16] Nakashima K, Itatani K, Kitamura T, Oka N, Horai T, Miyazaki S (2017). Energy dynamics of the intraventricular vortex after mitral valve surgery. Heart Vessels.

[17] Pridjian AK, Mendelsohn AM, Lupinetti FM, Beekman RH, Dick M, Serwer G (1993). Usefulness of the bidirectional Glenn procedure as staged reconstruction for the functional single ventricle. AM J Cardiol..

[18] Reddy VM, McElhinney DB, Moore P, Haas GS, Hanley FL (1997). Outcomes after bidirectional cavopulmonary shunt in infants less than 6 months old. J Am Coll Cardiol..

[19] Hayashi T, Itatani K, Inuzuka R, Shimizu N, Shindo T, Hirata Y (2015). Dissipative energy loss within the left ventricle detected by vector flow mapping in children: normal values and effects of age and heart rate. J Cardiol..

